# THE JOURNAL OF GERONTOLOGY: SOCIAL SCIENCES: GLOBAL SCHOLARSHIP CHALLENGES AND OPPORTUNITIES

**DOI:** 10.1093/geroni/igz038.705

**Published:** 2019-11-08

**Authors:** Deborah Carr

**Affiliations:** Boston University, Boston, Massachusetts, United States

## Abstract

Journal of Gerontology: Social Sciences aims to publish the highest quality social scientific research on aging and the life course in the U.S. and worldwide. The disciplinary scope is broad, encompassing scholarship from demography, economics, psychology, public health, and sociology. A key substantive focus is identifying the social, economic, and cultural contexts that shape aging experiences worldwide. In the coming decade, social gerontology research is poised to present many opportunities for cross-national and cross-cultural scholarship – driven in part by the proliferation of large parallel data sets from many nations in Europe, Latin America, and Asia. I will discuss the role that peer-reviewed cross-national scholarship can play in disseminating knowledge that informs gerontological research, policy, and practice internationally. I will also identify under-researched areas that will be of great interest to scholars in the coming decade, including LGBT older adults, aging in the Global South, reconfigured families, and centenarians.

